# Serogroup Conversion of Vibrio cholerae in Aquatic Reservoirs

**DOI:** 10.1371/journal.ppat.0030081

**Published:** 2007-06-08

**Authors:** Melanie Blokesch, Gary K Schoolnik

**Affiliations:** 1 Division of Infectious Diseases and Geographic Medicine, Stanford University School of Medicine, Stanford, California, United States of America; 2 Department of Microbiology and Immunology, Stanford University School of Medicine, Stanford, California, United States of America; Massachusetts General Hospital and Harvard Medical School, United States of America

## Abstract

The environmental reservoirs for Vibrio cholerae are natural aquatic habitats, where it colonizes the chitinous exoskeletons of copepod molts. Growth of V. cholerae on a chitin surface induces competence for natural transformation, a mechanism for intra-species gene exchange. The antigenically diverse O-serogroup determinants of V. cholerae are encoded by a genetically variable biosynthetic cluster of genes that is flanked on either side by chromosomal regions that are conserved between different serogroups. To determine whether this genomic motif and chitin-induced natural transformation might enable the exchange of serogroup-specific gene clusters between different O serogroups of *V. cholerae,* a strain of V. cholerae O1 El Tor was co-cultured with a strain of V. cholerae O139 Bengal within a biofilm on the same chitin surface immersed in seawater, and O1-to-O139 transformants were obtained. Serogroup conversion of the O1 recipient by the O139 donor was demonstrated by comparative genomic hybridization, biochemical and serological characterization of the O-antigenic determinant, and resistance of O1-to-O139 transformants to bacteriolysis by a virulent O1-specific phage. Serogroup conversion was shown to have occurred as a single-step exchange of large fragments of DNA. Crossovers were localized to regions of homology common to other V. cholerae serogroups that flank serogroup-specific encoding sequences. This result and the successful serogroup conversion of an O1 strain by O37 genomic DNA indicate that chitin-induced natural transformation might be a common mechanism for serogroup conversion in aquatic habitats and for the emergence of V. cholerae variants that are better adapted for survival in environmental niches or more pathogenic for humans.

## Author Summary

The reservoirs of Vibrio cholerae are aquatic environments, where it attaches to the chitin-containing shells of small crustaceans. Chitin serves as a nutrient for *V. cholerae* and it induces natural transformation, a process by which it acquires new genes from other microbes in the same habitat. The most compelling consequence of a V. cholerae gene acquisition event occurred in 1992 when a vast cholera epidemic erupted in India and Bangladesh and spread through Asia. Genetic analysis showed that this outbreak was due to the acquisition of a gene cluster that converted the ancestral V. cholerae O1 El Tor serogroup to an entirely new serogroup, designated O139 Bengal. This report shows that acquisition of the O139 gene cluster by an O1 El Tor strain can be mediated by natural transformation and that this can occur within a community of bacteria living on a chitin surface. The O139 derivatives of this transformation event were not killed by bacteriophages that attack O1 strains, explaining in part why O139 strains have replaced O1 strains in some Asian water sources. These results also illustrate how a combination of genetic and ecological factors can lead to the emergence of new pathogenic microbes in environmental reservoirs.

## Introduction

The reservoirs in nature for Vibrio cholerae, the cause of Asiatic cholera, are rivers, estuaries, and coastal waters, where it associates with the chitinous exoskeletons of copepod molts [1,2]. Chitin, a polymer of N-acetylglucosamine, not only serves as a surface for biofilm formation in aquatic habitats and as a nutrient source, but it also induces competence for natural transformation [3]. In the initial report of this phenomenon, competence was experimentally demonstrated by showing that it could mediate the acquisition of genes conferring antibiotic resistance during growth of a V. cholerae strain on a crab shell fragment immersed in seawater. This simple experimental system led to the identification of three positive regulators of the competence phenotype in *V. cholerae,* HapR, RpoS, and TfoX, and a type IV competence pseudopilus. These studies also showed that in addition to the chitin inducer, activation of the competence program required increasing cell density and declining nutrient availability, growth deceleration, or stress [3].

The use of antibiotic resistance markers to identify transformants carried with it the idea that competence could result in the acquisition of new genes to enhance genetic diversity. However, exactly which kinds of genes and functions might be acquired in this manner and how their acquisition could affect the evolution, ecology, or pathogenicity of this species was not addressed. In the work reported here, we begin to examine these issues by testing whether chitin-induced natural competence can mediate the uptake of genes that specify different V. cholerae O serogroups.

The V. cholerae species encompasses more than 200 serogroups [4]. Each serogroup is composed of a highly conserved lipopolysaccharide (LPS) lipid A and core region linked to a serogroup-specific O side chain, which projects from the outer membrane of the organism and whose antigenic character varies as a function of its monosaccharide composition, structure, and length. Until 1992, virtually all cases of endemic and epidemic cholera were due to the O1 serogroup of V. cholerae. Infection with V. cholerae O1 was found to stimulate an antibody response to the O1 side chain that was strongly correlated with the development of protective immunity [5,6].

In 1992, a heretofore unknown serogroup of V. cholerae was identified as the cause of cholera outbreaks in India and Bangladesh [7,8]. This historically unprecedented event was followed by its spread to other Asian countries, evoking concerns about a new cholera pandemic [9]. Molecular analysis of this serogroup, designated O139 Bengal [10,11], showed that a 22-kbp DNA segment encoding the O1 antigen (the *wbe* region) had been deleted from the ancestral O1 El Tor biotype and replaced by a 35-kbp DNA segment (the *wbf* region) specifying the new O139 serogroup antigen [12]. Unlike the O1-encoding region in El Tor strains, the O139-antigen gene cluster was found to specify not only the new O139 side chain, but in addition, a capsular polysaccharide (CPS) [13–15] that contains the same O139-antigenic determinant.

The close genetic relatedness of the ancestral O1 El Tor lineage with the new O139 serogroup [16–19], the existence of different O-antigen–encoding cassettes at the same chromosomal locus [20–23], and the presence of regions of homology on either side of this site [12,15,24,25] strongly support the hypothesis that some kind of horizontal gene transfer event had occurred, resulting in replacement of the original O1 gene cluster with the O139 gene cluster of a V. cholerae environmental strain [23,26–30]. However, neither the gene transfer mechanism nor the ecological context in which this occurred has been elucidated.

We show here that the O1-to-O139 gene cluster exchange can be mediated by chitin-induced natural transformation and that this can occur between two different V. cholerae serogroups living within the same biofilm on a natural chitin surface. These results have led us to hypothesize that this ecological niche and mechanism of horizontal gene transfer might provide a system for serogroup switching in this species.

## Results

### Chitin-Induced Natural Transformation Mediates Acquisition of the O139-Antigen–Encoding Region by an O1 V. cholerae Strain

**Figure 1:**
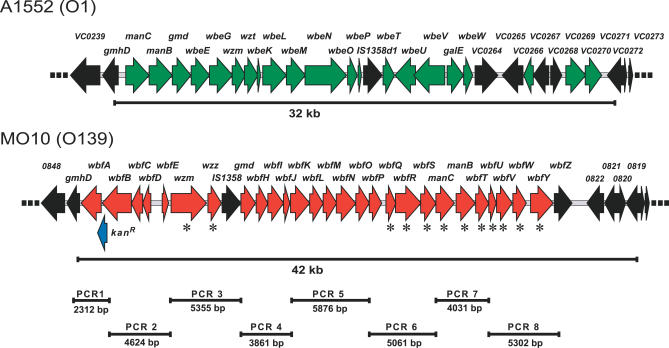
Comparison of the O-Antigen–Encoding DNA Region (wb*) of Strain A1552 (O1) and MO10 (O139) The genetic organization of the O1-specific and O139-specific DNA clusters on chromosome I of V. cholerae are shown (adapted from Heidelberg et al. [31], Stoeher et al. [28], Yamasaki et al. [33], Chatterjee and Chaudhuri [30], and the whole genome shotgun sequence of V. cholerae MO10). Genes and their orientation are denoted by arrows. Gene color code: green, O1-specific; red, O139-specific; black, homologous genes. The blue arrow depicts the Kan^R^ cassette (*aph* gene) integrated between *wbfA* and *wbfB* in strain VCO139-Kan. The bars below show the minimum size of the exchanged DNA fragments. The asterisks mark the 12 O139-specific genes present on the microarrays. PCR primers for the O139 region were designed to obtain the indicated O139-specific PCR fragments 1 through 8 shown at the bottom. ORFs: 0848 = VchoM_01000848 (homolog to VC0239), 0822 = VchoM_01000822 (homolog to VC0265), 0821 = VchoM_01000821 (homolog to VC0267), 0820 = VchoM_01000820 (homolog to VC0268), 0819 = VchoM_01000819 (homolog to VC0271).

To test if a V. cholerae O1 El Tor strain can acquire the O139-antigen–encoding region by natural transformation, a derivative of the O139 serogroup strain MO10 [21] was created by inserting a kanamycin resistance (Kan^R^) marker between the genes *wbfA* and *wbfB* within the O139 gene cluster (Figure 1). Designated VCO139-Kan, genomic DNA (gDNA) from this strain was used as the donor DNA in transformation experiments. V. cholerae O1 El Tor (strain A1552), previously shown to be competent for natural transformation during growth on a chitin surface [3], was used as the recipient and was propagated as a biofilm on a sterile crab shell fragment submerged in artificial seawater. gDNA from the VCO139-Kan donor was added to this biofilm culture; 24 h later, Kan^R^ transformants were selected on kanamycin-containing Luria-Bertani (LB) plates and counted, yielding a transformation frequency of 1.5 × 10^−5^. No transformants were detected in the absence of donor gDNA or if transformation-negative mutants (A1552*pilA* or A1552*hapR*) [3] were used as recipients.

**Figure 2:**
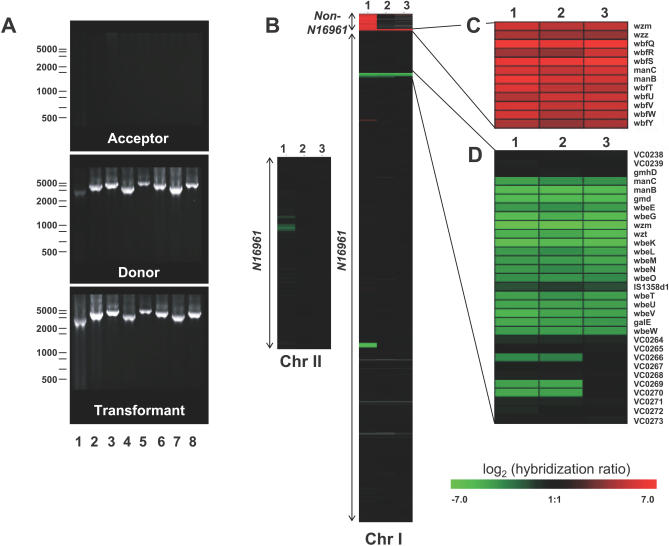
PCR and CGH Studies of Donor and Transformant gDNA Show Exchange of O-Antigen–Encoding Gene Clusters (A) gDNA from acceptor strain A1552 (O1), donor strain VCO139-Kan (O139 with Kan^R^ marker), and a representative transformant were used as template in a PCR experiment. Lanes: PCR fragments 1 to 8 spanning the whole O139-antigen–encoding region according to Figure 1. (B) CGH data. CGH was used to compare genes present in the V. cholerae O1 A1552 acceptor strain (labeled with Cy3) with genes absent/present in the donor strain VCO139-Kan (lane 1; labeled with Cy5) or two representative transformants of the acceptor strain (lanes 2 and 3; labeled with Cy5). Oligonucleotides corresponding to genes of the large chromosome (Chr I) and small chromosome (Chr II) of the sequenced strain N16961 [31] were spotted on the microarray (depicted as N16961) in addition to oligonucleotides derived from 12 O139-specific genes and from 82 genes corresponding to the SXT element (denoted as non-N16961; for details see Figure S5). (C and D) Close-up of the O139-antigen–specific genes (C) and of the O1-antigen–specific gene cluster (D).

About 15% of the Kan^R^ transformants (approximating a transformation frequency of 2.2 × 10^−6^) exhibited the same opaque colony morphotype as O139 strain MO10 [21] and were agglutinated by an O139 typing serum, but not by an O1 typing serum (Table 1). Thus, these opaque transformants of the V. cholerae O1 recipient express the O139-antigenic determinant. PCR assays using eight primer pairs that collectively span the entire 35-kbp O139-antigen–coding region (Figure 1; Table S1) showed that the O139-antigen–positive transformants had acquired the entire O139-antigen–encoding region (Figure 2A). Comparative genomic hybridization (CGH) using a microarray containing spots for all open reading frames (ORFs) of the V. cholerae O1 El Tor sequenced strain N16961 [31] and for 12 genes within the O139-antigen−coding region (Figure 1) demonstrated that the transformants were identical to the O1 El Tor recipient (Figure 2B), except for the absence of the O1-antigen–encoding gene cluster (Figure 2D) and the presence of the 12 assayed O139-antigen–specific genes (Figure 2C).

**Table 1:**
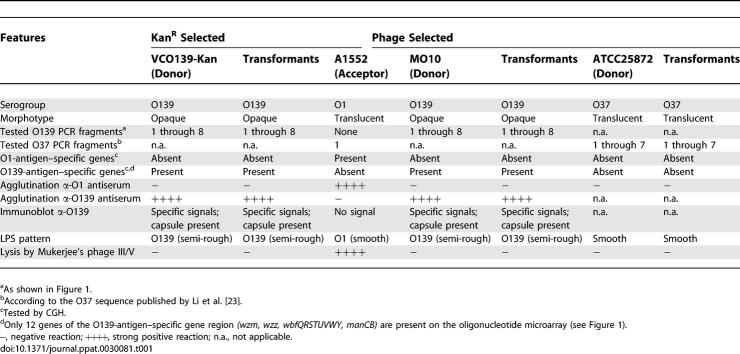
Genotype and Phenotype of the Transformants

The location of crossovers between the recipient genome and the transforming donor DNA was deduced from the CGH results. In most of the O139-antigen–positive transformants, the microarray results localized crossovers to regions of homology flanking the O-antigen gene cluster, within or upstream of *gmhD* at the left junction (Figure 2D, lanes 2 and 3), and within or downstream of VC0271 at the right junction (Figure 2D, lane 2). However, in a few transformants, crossovers could be localized to a different right-junction site within a second region of homology spanning the ORFs VC0264–VC0265 (Figure 2D, lane 3). Because all of the identified crossover sites were within regions of homology, the exchange of O-antigen–encoding cassettes was likely mediated by homologous recombination.

The residual 85% of the Kan^R^ transformants had a rough LPS phenotype. We analyzed their genotype and found that all of them had undergone a homologous recombination event with one crossover within or upstream of the *gmhD* gene (similar to the transformants having the O139 phenotype); a second crossover was detected inside the IS*1358d1* and IS*1358* genes of the O1 and O139 strains, respectively. This observation raised the possibility that a homologous recombination event at this site is required as the first of two steps in the acquisition of the entire O139-coding region. This issue was addressed in the experiments described below.

### The Entire O139-Antigen–Encoding Region Is Exchanged by a Single Transformation Event

Between the homologous regions described above, which flank the O-antigen–encoding clusters of serogroups O1 and O139, the only other locus of significant homology encompasses two orthologous genes with 96% sequence identity: IS*1358d1* (within the O1-antigen cassette) and IS*1358* (within the O139 cassette; Figure 1). To determine if this internal region of homology also provides a site for homologous recombination [27], IS*1358* was deleted in the Kan^R^ O139 donor strain (strain VCO139-KanΔIS1358) and its purified gDNA used in transformation experiments. Kan^R^ opaque transformants, acquired at a frequency of 1.3 × 10^−6^, were found by PCR analysis and CGH to be identical to O139-antigen–positive transformants obtained through the use of gDNA containing an intact IS*1358* gene. This shows that the internal IS*1358* site of homology is not required for serogroup transformation. It follows that O1-to-O139 serogroup conversion likely occurs as a single transformation event and entails the exchange of very large fragments of DNA: ≥ 32-kbp of sequence containing the O1-antigen–coding region was exchanged for ≥ 42-kbp of sequence containing the O139-antigen–coding region.

### O1-to-O139 Transformants Produce an O139 LPS, Capsule, and Antigenic Determinant

**Figure 3:**
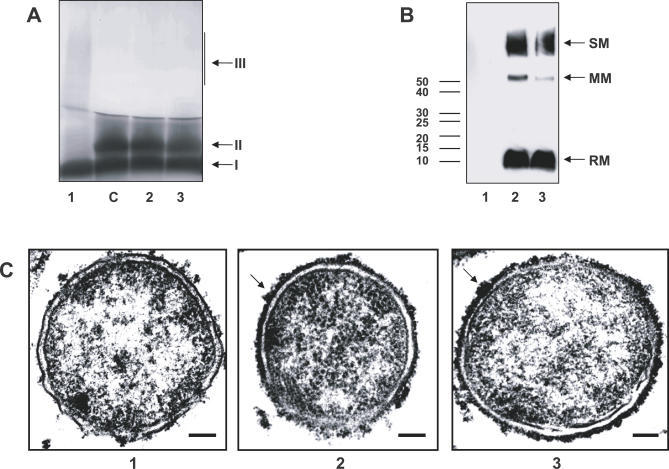
O1-to-O139 Transformants Produce O139 LPS and Antigenic Determinant and Are Encapsulated The acceptor strain A1552 (lane 1), the donor strain VCO139-Kan (lane 2), and a representative transformant (lane 3) were analyzed for LPS structure and for the presence of CPS and O-antigenic determinants. (A) LPS analysis. Purified LPS was subjected to SDS-PAGE (15% gel) and stained with silver. Specific signals, by arrows, depict the lipid A-core (I), the lipidA-core-O139 semi-rough LPS (II), and the lipidA-core-O1 LPS (III). The O139 parental strain MO10 [21] is shown in lane C. (B) O139-specific immunoblot analysis. Cell-lysates were separated in a 15% SDS gel, transferred onto a polyvinylidene fluoride membrane, and reacted with α-O139 polyclonal antibodies. Capsule-specific bands (SM, slowly migrating; MM, medium migrating) and O-antigen–specific bands (RM, rapidly migrating [14]) are indicated by arrows. (C) Detection of the CPS by electron microscopy. Cells were treated with polycationic ferritin and the CPS then visualized by transmission electron microscopy. The location of the capsule is indicated by arrows. Scale bar: 0.1 μm.

The O1 and O139 serogroups have distinctive LPS structures and O-antigenic determinants. To discover whether O1-to-O139 transformants produce an O139 LPS and CPS typical of the O139 donor strain, LPS was isolated from the O139 control strain MO10, the O139 donor strain VCO139-Kan, and O139-antigen–positive transformants, and studied by SDS-PAGE and silver staining. LPS from each strain and from the transformants showed identical banding patterns: all exhibited the short O-antigen side chain that typifies O139 isolates (semi-rough LPS; Figure 3A). In addition, the presence of a CPS carrying the O139-antigenic determinant was detected in immunoblot assays using an O139-specific antiserum (Figure 3B). Encapsulation of O139 transformants was conclusively demonstrated by ultrastructural studies of polycationic ferritin–stained thin sections (Figure 3C). Thus, with respect to encapsulation, LPS structure, and antigenic specificity, the O139 donor and the transformants were identical (Table 1).

### Serogroup-Converted Transformants Are Not Killed by an O1-Specific Bacteriophage

Acquisition of the O139-antigen gene cluster by the ancestral O1 El Tor strain seems to have favored the recipient's growth and amplification in humans already immune to the O1 serogroup, and likely explains its emergence as a major cause of cholera. The basis by which it continues to persist within environmental reservoirs is more obscure, but this persistence suggests that the O139 cassette also confers a fitness advantage in some environmental niches. One such advantage might be the capacity to evade predation by phages.

**Figure 4:**
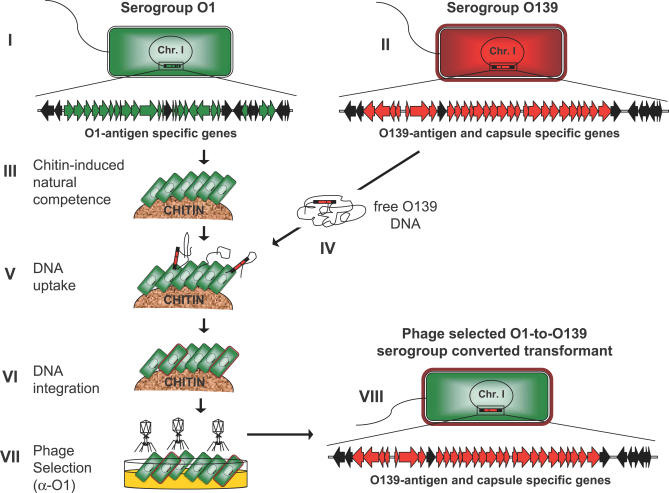
Schematic Representation of V. cholerae Serogroup Conversion by Chitin-Induced Transformation and Phage-Mediated Selection V. cholerae serogroups O1 (I) and O139 (II) are shown as green and red bacteria, respectively. They differ in the size and composition of the O-antigen moiety of LPS and by the presence of a polysaccharide capsule on the surface of the O139 serogroup. These differences are encoded by the O-antigen–specific gene cluster depicted below each bacterium. Black arrows (I, II, and VIII) denote the location of homologous genes present in both serogroups (for detail, see Figure 1). V. cholerae O1, grown on a chitin surface, becomes competent for transformation (III). Free DNA from the O139 donor strain (IV) is taken up (V) and integrated into the chromosome by homologous recombination (VI). Exchange of the O1 gene cluster for the O139 cluster leads to serogroup conversion. The transformed recipient produces the O139 O antigen and CPS (VIII) and becomes resistant to O1-specific bacteriophages (VII).

To test this possibility and simulate a possible scenario for the evolution of the O139 Bengal epidemic lineage, we repeated the transformation experiment without antibiotic selection (Figure 4). We established a biofilm of the O1 El Tor strain on a chitin surface and used gDNA from the O139 strain MO10 [21] as donor DNA, which lacks an antibiotic selectable marker within the O139-specific region. The resulting O139 transformants were selected through the use of a virulent bacteriophage (Mukerjee's El Tor phage V; [32]) which lyses the O1 serogroup strain A1552, but not the O139 strain MO10 (Figure 4). Surviving opaque transformants were characterized and found to be identical to the O139 donor with regard to LPS structure, antigenic specificity, and encapsulation (Table 1). PCR analysis and CGH demonstrated that the phage-selected transformants harbor an exchanged O-antigen gene cluster (O1 deleted, O139 gained; Figure S1), but are otherwise genetically identical to the O1 recipient and distinguishable from the donor strain M010 (Figure S5).

### O-Antigen Gene Exchange between Different V. cholerae Serogroups in a Biofilm Community on a Chitin Surface

In the experiments described above, purified gDNA prepared from an O139 donor was added to a monoculture of the A1552 strain of V. cholerae O1 growing on a chitin surface. However, most biofilm communities in natural aquatic habitats are composed of taxonomically diverse assemblages of microorganisms, thus providing an opportunity for the exchange of genetic material between different strains and species. To test whether different serogroups of V. cholerae can exchange serogroup-specific coding sequences when co-cultivated on a chitin surface, we grew a V. cholerae O1 strain and a V. cholerae O139 strain on the same crab shell fragment for 2 d without externally added DNA (Figure S2). The two strains could be distinguished by their antibiotic resistances: the O1 strain A1552 is rifampicin resistant (Rif^R^) and streptomycin sensitive (Strep^S^), whereas the O139 strain VCO139-Kan is Strep^R^ and Rif^S^ and carries the Kan^R^ marker within the O139 gene cluster. Then, bacteria were harvested from the crab shell surface and the O1-to-O139 serogroup transformants of the O1 strain A1552 were selected on LB plates containing rifampicin and kanamycin and verified to be sensitive to streptomycin. Quantitative analysis of the results yielded a transformation frequency of 6.0 × 10^−7^. If DNase was added to this surface-attached consortium, no Rif^R^, Kan^R^, Strep^S^ colonies were obtained, thereby excluding conjugation and transduction as the mechanism of horizontal gene transfer in this experiment. The exchange of the O-antigen–encoding cluster (O1 cluster deleted, O139 cluster inserted) was confirmed by PCR analysis and CGH (Figure S3). Thus, natural transformation can cause serogroup conversion within biofilm communities composed of different V. cholerae serogroups.

### Chitin-Induced Natural Transformation Mediates O1 Conversion to a Non-O139 Serogroup

Sequences flanking the O-antigen gene cluster are not only homologous between the O1 and O139 serogroups [12,15], but they also are homologous in several non-O1/non-O139 V. cholerae serogroups [25,33]. This conserved pattern—regions of homology on either side of a region of difference—provides a conserved genomic motif that might enable the exchange of serogroup-specific gene clusters between many of the more than 200 different O serogroups of V. cholerae. To test this prediction, we used chitin-induced natural transformation to transform the A1552 O1 serogroup strain with gDNA from an O37 serogroup strain (ATCC25872; [34]) that caused a localized outbreak of diarrhea in Czechoslovakia in 1965 [35]. We then used an O1-specific lytic phage to select transformants of the A1552 strain that lack the O1 determinant, using the phage selection method discussed above. The acquisition of O37-specific genes by the A1552 transformants, together with the deletion of the O1-antigen gene cluster, was demonstrated by PCR analysis and CGH, respectively (Table 1; Figure S4). Using the antibiotic-based screening method described above and donor DNA from a V. cholerae strain harboring a Kan^R^ marker inside the O37-specific gene region (strain O37-Kan; Protocol S1) we computed a transformation frequency of 2.3 × 10^−6^. These results suggest that the conversion of O1 serogroup strains by transformation-dependent acquisition of other O-serogroup–encoding cassettes may occur commonly in aquatic habitats where different V. cholerae lineages co-exist.

## Discussion

In this report, we show that chitin-induced natural transformation can mediate the serogroup conversion of a V. cholerae O1 epidemic strain via the acquisition of an O139-antigen–encoding cassette. We further show that transformation-dependent serogroup conversion can proceed as a single step and results in the exchange of very large fragments of DNA. Finally, we demonstrate that this can occur on a natural chitin surface within a biofilm composed of the donor and recipient strains and without the need of exogenously added DNA. The resulting transformants have the genetic background of the O1 recipient, but produce an LPS and capsule indistinguishable from that of the O139 donor.

These results indicate that chitin-induced natural transformation could have been the mechanism of horizontal gene transfer responsible for the appearance of the O139 Bengal serogroup in 1992. However, a laboratory-based demonstration of this kind conducted in the present era does not necessarily recapitulate events that occurred in the past in an environmental setting. Thus, our results cannot exclude the possibility that the original serogroup conversion event was due to another mechanism of horizontal gene transfer, e.g., a transducing phage or conjugative plasmid, both of which could carry sufficiently large fragments of DNA into the recipient genome.

Unlike transducing phage or conjugative plasmids, transformation does not require the intercession of mobile genetic elements. Consequently, transformation can, in principle, mediate the transfer of fragments from any part of the genome. Because such fragments are ordinarily incorporated into the recipient chromosome by homologous recombination, transformation usually mediates gene exchange between taxonomically closely related organisms and especially between strains of the same species. For genetically diverse species such as V. cholerae [36–41], fragments acquired by transformation can be expected to affect fitness through the acquisition of genes that are present in the donor but missing or defective in the recipient, and whose presence in the recipient would confer a selective advantage [42]. One such gene class, which varies between V. cholerae serogroups, is the O-antigen–encoding sequences, which, based on antigenic differences, exceed 200 in number [4]. Were each of these O-antigen–encoding cassettes to reside between highly homologous regions, then in principle natural transformation could cause serogroup conversion of an O1 strain to any other of the ~200 V. cholerae serogroups. To investigate this possibility, we surveyed the O-antigen flanking regions of available V. cholerae sequences from the *Vibrio* genome project at The Institute for Genomic Research (whole genome shotgun sequences). These include representatives of the O12, O37, O39, O135, O139, O141, and O1 classical serogroups. The flanking sequences of these O-antigen–coding regions were then compared to the V. cholerae O1 El Tor N16961 flanking sequences [31] and found to be highly homologous ( ≥90%). This analysis, while limited to a small fraction of the extant V. cholerae O-antigen regions, supports the idea that exchange of the O-antigen genes could potentially occur between large numbers of V. cholerae serogroups. This idea is reinforced by the work of others showing that the same V. cholerae lineage can produce different O antigens and, conversely, that different V. cholerae lineages can produce the same O antigen [23,43,44]. These data and the successful conversion of an O1 strain by O37 gDNA (Table 1; Figure S4) suggest that different O-antigen gene cassettes might be able to recombine freely within and between different V. cholerae lineages.

However, the prediction that O-antigen cassettes are frequently exchanged between different V. cholerae lineages is not supported by serogroup determinations of V. cholerae isolates from patients with cholera: only the O1-to-O139 conversion event has led to the emergence of a new pathogenic variant with the capacity to spread regionally, persist within environmental reservoirs, and cause periodic outbreaks many years after it first appeared. A comprehensive study by Li et al. underscores this point [23]. They screened 300 V. cholerae strains representing 194 different non-O1/non-O139 serogroups for cholera toxin genes and virulence genes within *Vibrio* pathogenicity island 1, and they used genotyping methods to identify strains with the O1 genetic background. Only four non-O1/non-O139 serogroups (O27, O37, O53, and O65) were found that possessed the virulence genes and genotype typical of most V. cholerae O1 and O139 strains [23]. Thus, of 194 V. cholerae serogroups studied, only four serogroups were identified that appear to have been derived from an O1 progenitor by serogroup conversion at the O-antigen (wb*) region. By contrast, analysis of O139 strains isolated from different sites and in different years showed that the O139-encoding cassette had been acquired by genotypically different progenitors via multiple separate horizontal gene transfer events since the first O139 serogroup was detected in 1992 [45–47]. Thus, while the analysis of homologous flanking regions indicated that serogroup switching between O1 stains and strains of other serogroups might occur commonly, this does not seem to have occurred in nature except for the O1-to-O139 serogroup conversion event.

A possible resolution of this paradox comes from a careful examination of the ecological context and necessary prerequisites for transformation-dependent serogroup conversion. Four factors are required: the presence of a recipient V. cholerae capable of being induced to a state of competence by chitin; the inducing polysaccharide (chitin); a source of donor DNA provided by a different V. cholerae serogroup living in association with the recipient on the same chitin surface; and the clonal expansion of the resulting serogroup-converted transformant. From this list of prerequisites it is possible to identify barriers and bottlenecks that would limit the exchange of serogroup cassettes. First, not all V. cholerae strains can be induced to competence by chitin [3]. In some non-competent strains this is due to mutations in *hapR* [48,49] which encodes a positive regulator of competence [3] and a negative regulator of virulence [48]. Although mutants of this kind do not exhibit chitin-induced competence, they might be relatively common during cholera outbreaks because their hyper-virulent phenotype could confer a selective advantage during in vivo growth. Second, it is unclear how frequently two or more V. cholerae serogroups occupy the same chitin surface in sufficiently close proximity that DNA from one would be accessible to the other. Indeed, co-occupation of the same niche would seem to violate the niche exclusion principle, which holds that no more than one species or genotype can occupy the same niche (in this instance, a particular chitin surface), especially if one multiplies more rapidly than the other [50]. Whether this would apply to two or more V. cholerae serogroups on the same chitin surface has not been tested by competition experiments. Third, the availability of chitin, the inducing substrate, likely varies as a function of season, being abundant during copepod blooms and less so at other times. Finally, not all serogroup-converted transformants will enjoy a selective advantage compared to the ancestral O1 recipient strain. Without a gain in fitness in some important environmental or in vivo niche, clonal expansion of the transformant is unlikely to occur. That said, serogroup conversion between non-O1/non-O139 environmental isolates of V. cholerae could occur commonly and might confer a selective advantage in aquatic habitats. However, such strains, lacking pathogenicity determinants, would be under-represented in most strain collections, which are biased for strains isolated from human cases of diarrhea.

There is compelling evidence that the O1-to-O139 serogroup conversion event conferred a selective advantage in environmental reservoirs and the human gastrointestinal tract. First, as shown in this report, an O1-to-O139 serogroup–converted transformant was found to be resistant to an O1 lytic phage, a finding suggesting that serogroup switching could be a mechanism for avoiding phage predation in aquatic habitats. This laboratory demonstration is supported by the observation of Faruque and colleagues, who used water samples collected in Bangladesh to show that the concentration of *Vibrio* phages in the environment is inversely correlated with the concentration of the V. cholerae host, leading them to hypothesize that lytic phage might be responsible for the termination of cholera outbreaks and for the emergence of new, phage-resistant V. cholerae variants [51]. In addition, V. cholerae O139 variants form thicker biofilms on abiotic surfaces than O1 strains [52], another trait that could enhance survival of O139 strains in aquatic habitats. The rapid spread of the O139 serogroup through communities in India and Bangladesh and its capacity to infect adults and children alike has been attributed to its infectivity for persons previously immune to the ancestral O1 serogroup [11]. In addition, O139 strains have been shown to colonize the suckling mouse model of cholera more efficiently than O1 strains [14]. Thus, clonal expansion of O139-serogroup convertants seems to have occurred because the O139 side chain and capsule confer a selective advantage in niches of the organism that are critical for its success, both as an infectious agent of humans and as a microbe indigenous to aquatic habitats.

## Materials and Methods

### Bacterial strains and growth conditions.

Bacterial strains used in this study are V. cholerae A1552 (serogroup O1 El Tor [53]), MO10 (serogroup O139 [21,54]), VCO139-Kan (MO10 with Kan^R^ cassette inserted between *wbfB* and *wbfA*), O139-KanΔIS*1358* (VCO139-Kan, deletion in IS*1358*), and ATCC25872 (serogroup O37 [34,35]). V. cholerae was grown either in LB medium or in defined artificial seawater medium.

### Transformation on crab shell surfaces.

Transformation on crab shell surfaces as a monoculture or in mixed biofilm communities was performed at least in triplicate as described [3]. Details of the protocol can be found online as Protocol S1.

### PCR analysis of transformants.

gDNA was isolated using Qiagen kits (http://www.qiagen.com) and used as template for polymerase chain reaction (PCR). The PCR was performed as recommended using the HotStarTaq Master Mix Kit (Qiagen). The priming positions of the O139-specific oligonucleotides are shown in Figure 1; the O37-specific primers were designed according to the O37 sequence published by Li et al. [23]. All primer sequences are listed in Table S1.

### CGH.

For CGH, 1 μg gDNA was labeled using the BioPrime Array CGH Genomic Labeling kit (Invitrogen, http://invitrogen.com) and Cy3 or Cy5 as dyes. The samples were hybridized to oligonucleotide DNA microarrays designed according to the ORFs of V. cholerae O1 El Tor strain N16961 [31]. In addition, 12 oligonucleotides representing genes of the V. cholerae O139-antigen region (Figure 1) are spotted on the array. Microarray raw data (see Table S2) are deposited at the Stanford Microarray Database (http://smd.stanford.edu) and the National Institutes of Health (NIH) National Center for Biotechnology Information's Gene Expression Omnibus (GEO) (http://www.ncbi.nlm.nih.gov/projects/geo). CGH was performed in duplicate for each biological sample.

### LPS preparation and silver staining.

Isolation of LPS was performed as described [55], separated by SDS– polyacrylamide gel electrophoresis (PAGE) and visualized by silver staining [56].

### Immunoblot analysis.

Whole cell-lysates were separated by SDS-PAGE, electroblotted onto PVDF membranes, and reacted with O139-antigen–specific antibodies (Remel, http://www.remelinc.com). For signal detection, Protein A-HRP conjugate (Bio-Rad, http://www.bio-rad.com) and Lumi-Light Western Blotting substrate (Roche Applied Science, http://www.roche-applied-science.com) were used.

### Electron microscopy.

The polysaccharide capsule was visualized by polycationic ferritin treatment and transmission electron microscopy [57,58].

### Slide agglutination tests.

Slide agglutination tests using polyclonal α-O1 and α-O139 antisera, respectively, were performed as suggested by the manufacturer (BD Difco [http://www.bd.com] and Remel).

### Phage procedures.

Phage lysates of Mukerjee's phages [32] were a gift of M. S. Islam. After the transformation experiment, bacteria were detached from the chitin surface, added to soft-agar, and overlaid onto LB plates. Phage-mediated selection was performed by dropping Mukerjee's El Tor phage III and V [32], respectively, onto this bacteria-containing soft-agar. After an 18- to 24-h incubation period at 37 °C, resistant bacteria from the plaques were picked and further analyzed. Transformation experiments followed by phage-mediated selection were performed in four independent experiments.

## Supporting Information

Figure S1 Phage-Mediated Selection Leads to Accumulation of Serogroup-Converted Transformants (A) gDNA of acceptor strain A1552 (O1), donor strain MO10, or a representative transformant was used as template in a PCR experiment. Lanes: PCR fragments 1 to 8 spanning the whole O139-antigen–encoding region according to Figure 1.
(B and C) Present/absent genes of the O1- and O139-specific gene cluster. Acceptor strain A1552 was labeled with Cy3, and the donor strain MO10 (lane 1) or two representative transformants (lanes 2 and 3) were labeled with Cy5 and compared by CGH. O1-specific genes are shown in (B), O139-specific genes in (C).
(104 KB PDF)

Figure S2 Schematic Representation of V. cholerae Serogroup Conversion by Chitin-Induced Transformation in Mixed Biofilm Communities V. cholerae serogroups O1 (I) and O139 (II) are shown as green and red bacteria, respectively (for detail, see Figure 4). V. cholerae O1, co-cultured with strain O139 (III), becomes competent for transformation on a chitin-surface (IV). Released DNA from lysed bacteria (V) of the O139 donor strain is taken up and integrated into the chromosome by homologous recombination (VI). Antibiotic selected transformants (Rif^R^, Kan^R^, Strep^S^), which exchanged their O1 gene cluster for the O139 cluster, produce the O139 O-antigen and CPS (VIII). If DNase is added (III to VI), no transformants (VIII) can be selected.
(81 KB PDF)

Figure S3 Serogroup Conversion Occurs in Mixed Biofilms (A) gDNA of acceptor strain A1552 (O1), donor strain VCO139-Kan (O139 with Kan^R^ marker), and a representative transformant were used as template in a PCR experiment. Lanes: PCR fragments 1 to 8 spanning the whole O139-antigen–encoding region according to Figure 1.
(B and C) Comparison of the presence/absence of O1- and O139-specific genes by CGH. Acceptor strain A1552 was labeled with Cy3, and the donor strain VCO139-Kan (lane 1) or a representative transformant (lanes 2) was labeled with Cy5. O1-specific genes are shown in (B), O139-specific genes in (C).
(82 KB PDF)

Figure S4 PCR and CGH Analysis of Donor and Transformant gDNA Show Exchange of O-Antigen–Encoding Gene Clusters (A) gDNA of acceptor strain A1552 (O1 serogroup), donor strain ATCC25872 (O37 serogroup), or a representative transformant was used as template in a PCR experiment. Lanes: PCR fragments 1 to 7 spanning the whole O37-antigen–encoding region according to the published O37 sequence of Li et al. [23].
(B) CGH data. Comparison of absent/present genes in the donor strain ATCC25872 (lane 1; labeled with Cy5) or a representative transformant (lane 2; labeled with Cy5) to the acceptor strain A1552 (labeled with Cy3). Oligonucleotides present on the microarray correspond to genes of the large chromosome (Chr I) and small chromosome (Chr II) of the sequenced strain N16961 [31].
(C) Close-up of the O1-antigen–specific gene cluster.
(126 KB PDF)

Figure S5 CGH of DNA Regions That Differ between Strains A1552 and MO10 Absent/present genes in the donor strains VCO139-Kan and MO10 (lanes 1 and 5, respectively; labeled with Cy5) or the transformants (Kan^R^ selected, lanes 2 and 3; Kan^R^ selected after mixed biofilm experiment, lane 4; phage selected, lanes 6 and 7) in comparison to the acceptor strain A1552 (lanes 1 to 7; labeled with Cy3).
(A) Gene cluster VC0510–0515.
(B) Part of the integron on the small chromosome [59].
(C) Part of *Vibrio* pathogenicity island 2 spanning genes VC1761–VC1788 (as described earlier [60,61]).
(D) Genes of the SXT element [62]. None of the O1-to-O139 transformants has gained any of these DNA regions and therefore are readily distinguishable to the donor strains.
(436 KB PDF)

Protocol S1 Supporting Methods (137 KB PDF)

Table S1 Oligonucleotides Used in This Study (119 KB PDF)

Table S2 List of Microarrays and Corresponding GEO Accession Numbers (75 KB PDF)

### Accession Numbers

The microarray data discussed in this publication have been deposited in the Stanford Microarray Database (http://smd.stanford.edu) and the NIH National Center for Biotechnology Information's (NCBI) GEO (http://www.ncbi.nlm.nih.gov/projects/geo) and are accessible through GEO Series accession numbers GSE5120, GSE5302, GSE5932, GSE5934, and GSE5935 (SuperSeries GSE5303). The NCBI (http://www.ncbi.nlm.nih.gov) accession number for the whole genome shotgun sequence of V. cholerae MO10 is NZ_AAKF00000000.

## Abbreviations

CGH: comparative genomic hybridization
CPS: capsular polysaccharide
gDNA: genomic DNA
Kan^R^: kanamycin resistance
LPS: lipopolysaccharide
ORF: open reading frame
Rif^R^: rifampicin resistant
Strep^S^: streptomycin sensitive
