# Supplementary material for: Serogroup Conversion of Vibrio cholerae in Aquatic Reservoirs
Source: PLoS Pathog. 2007 Jun 8;3(6):e81. doi: 10.1371/journal.ppat.0030081 (PMC1891326; doi:10.1371/journal.ppat.0030081)
Supplement: Supplementary file 1 [file ppat.0030081.sd001.pdf]

Supporting Information

Serogroup conversion of *Vibrio cholerae* in Aquatic Reservoirs

Melanie Blokesch and Gary K. Schoolnik

Supporting Material and Methods

Strain constructions.

Strain VCO139-Kan is a derivative of *Vibrio cholerae* O139 Bengal MO10 [1,2]. To insert the Kanamycin resistant cassette into the O139 specific region, the *aph* gene (amino-glycoside phosphotransferase) was amplified by PCR with the oligonucleotides 3-kanR-up-OL-wbfB and 4-kanR-down-OL-wbfA (Table S1) using plasmid pET28a (Novagen, Madison, WI) as template. Parts of the genes *wbfA* and *wbfB* were amplified with primers 1-wbfB-up-NcoI / 2-wbfB-end-OL-Kan and 5-wbfA-start-OL-Kan / 6-wbfA- down-SacI (Table S1), respectively, using gDNA from strain MO10 as template. The PCR products of these reactions were purified (Qiagen PCR purification kit) and used as templates in an overlapping-primer PCR (Primers: 1-wbfB-up-NcoI and 6-wbfA-down-SacI; Table S1). The resulting gel-purified PCR fragment (end of *wbfA* gene, Kan^R gene *aph*, beginning of *wbfB* gene) was digested with *Nco*I and *Sac*I (New England Biolabs) and ligated into the counter selectable plasmid pGP704-Sac28 [3] (*Nco*I and *Sac*I cut). The plasmid was introduced into *Vibrio cholerae* strain MO10 [1] by biparental mating with *E. coli*. Sucrose-based counter selection was done as described [4]. Introduction of the *aph* gene was checked by resistance to Kanamycin and confirmed by

PCR and sequencing. Strain VCO139-Kan and its parental strain MO10 were found to be indistinguishable with respect to LPS pattern in silver stained SDS gels and the presence and appearance of the capsular polysaccharide (ascertained by immunoblot analysis and electron microscopy).

Strain VCO139-KanΔIS1358 is a derivative of VCO139-Kan. The deletion of the IS*1358* gene was accomplished by the method described [3] using the oligonucleotides 1-IS1358-KO-SacI / 2-IS1358-KO and 3-IS1358-KO / 4- IS1358-KO-NcoI (Table S1) and gDNA from strain MO10 [1] as template for the PCR. Its accuracy was checked by PCR and confirmed by sequencing.

Strain O37-Kan is a derivative of ATCC25873 [5,6]. The kanamycin resistant gene was inserted between ORF6 and ORF7 in the O37 specific region [7]. Therefore the flanking regions (end of ORF6 and beginning of ORF7) were amplified using the primer pairs 1-ORF6 -up-*XbaI*, 2-ORF6-end-OL-Kan and 5-ORF7-start-OL-Kan, 6-ORF7-*down*-*Sac*I, respectively. The Kan^R cassette was gained from a PCR reaction with pET28a (Novagen, Madison, WI) as template and 3-*kanR*-up-OL-ORF6 and 4-*kanR*-down-OL-ORF7 as primers (Table S1). The fragments were joined by an overlapping PCR using the oligonucleotides 1-ORF6 -up-*XbaI* and 6-ORF7-*down*-*Sac*I. The resulting PCR fragment was gel purified and directly used as donor DNA in a chitin-dependent transformation experiment. The resulting strain was analyzed by PCR using seven primer pairs that collectively span the entire O37-antigen coding region (see Table S1), by sequencing and CGH.

Detailed protocol for transformation on crab-shell surfaces

Vibrio cholerae acceptor strains (A1552 derivatives) were grown aerobically in LB broth (250 rpm) until they reached an OD_{600} ~ 0.2-0.3 [8]. The bacterial cultures were harvested by centrifugation, washed with defined artificial seawater media (0.25 X DASW: 117 mM NaCl, 13.75 mM $MgSO_4$, 0.75 mM $NaHCO_3$, 2.475 mM $CaCl_2$, 2.575 mM KCl, 0.035 mM $Na_2B_4O_7$, 0.025 mM SrCl, 0.0075 mM NaBr, 0.0005 mM NaI, 0.0065 mM LiCl, 9.35 mM NH_4Cl, 0.0935 mM K_2HPO_4, 25 mM HEPES, pH 7.4.) and resuspended in twice the culture volume of the same media supplemented with vitamins. Aliquots of 2 ml of this bacterial suspension were used as inoculum of sterile crab-shell pieces (approximate surface area of 1.2 cm^2) in a 12-well plate. A biofilm was allowed to form on the crab shell surface at 30ºC between 18-24 hours post-inoculation. Subsequently, planktonic bacteria and media were removed and fresh media (2 ml DASW + vitamins) was added. Immediately after this medium exchange, 2 µg gDNA from the donor strain was added (4µg gDNA in the case of the phage-selected experiment). The bacteria were grown for an additional 24 hours, washed and detached from the crab-shell fragments by vortexing for 30 sec. Undiluted and diluted *V. cholerae* cells were plated onto LB plates without antibiotic and onto antibiotic-containing LB plates (75µg/ml kanamycin). The transformation frequency was calculated as the ratio of antibiotic resistant colony forming units (CFU) against total CFUs.

Transformation on crab-shell surfaces in a mixed biofilm community

Cultures of *V. cholerae* A1552 and VCO139-Kan (MO10 [1] derivative harboring a Kan^R marker within the O139 gene cluster) were grown in LB medium (until OD_{600} ~ 0.3). The

bacteria were harvested, washed and resuspended in defined artificial seawater medium as described above. One ml bacterial suspensions of each strain were mixed and added to a well containing a sterile piece of crab-shell. After incubation at 30ºC for 24 hours, medium containing planktonic bacteria was removed and fresh DASW medium was added. 24 hours later, the bacteria were washed and detached from the crab shell fragment. As a control the same experiments were performed in the presence of DNase (50 U; added four times within the two day incubation period). Released bacteria were plated onto LB plates and LB plates supplemented with antibiotics. The A1552 acceptor strain is Rifampicin resistant (Rif^R), VCO139-Kan donor strain is Streptomycin and Kanamycin resistant ($Strep^R$, Kan^R) and transformants which gained the O139 gene cluster are Rif^R and Kan^R, but Streptomycin sensitive (see Fig. S2). Transformation frequencies are calculated from at least three independent experiments.

Remarks for transformation on crab-shell surfaces

Not all *Vibrio cholerae* strains can become competent for natural transformation. Quorum-sensing is involved in the regulation circuit of the competence program [8]. HapR, a regulator of quorum-sensing, is defective in some laboratory strains as its gene possesses a frameshift mutation [9] or point mutation with a deleterious effect on the function of HapR [10]. Strains harboring mutations of this kind will not exhibit chitin-induced natural transformation. One such strain is the fully sequenced *V. cholerae* O1 El Tor strain N16961; complementation of this strain with a wild type copy of *hapR* restores the competence phenotype [8]. However, we have found that many other *V. cholerae* strains are naturally competent during growth with chitin including A1552 (used in this

study), ATCC25872, ATCC25873 (Melanie Blokesch, unpublished results) and *V. cholerae* isolates from the California coast (Miller *et al*.; submitted).

Supporting references:

1. Waldor MK, Mekalanos JJ (1994) *Vibrio cholerae* O139 specific gene sequences. Lancet 343: 1366.
2. Waldor MK, Mekalanos JJ (1994) ToxR regulates virulence gene expression in nonO1 strains of *Vibrio cholerae* that cause epidemic cholera. Infect Immun 62: 72-78.
3. Meibom KL, Li XB, Nielsen AT, Wu CY, Roseman S, et al. (2004) The *Vibrio cholerae* chitin utilization program. Proc Natl Acad Sci USA 101: 2524-2529.
4. Fullner KJ, Mekalanos JJ (1999) Genetic characterization of a new type IV-A pilus gene cluster found in both classical and El Tor biotypes of *Vibrio cholerae*. Infect Immun 67: 1393-1404.
5. Aldova E, Laznickova K, Stepankova E, Lietava J (1968) Isolation of nonagglutinable vibrios from an enteritis outbreak in Czechoslovakia. J Infect Dis 118: 25-31.
6. Felsenfeld O, Stegherr-Barrios A, Aldova E, Holmes J, Parrott MW (1970) In vitro and in vivo studies of streptomycin-dependent cholera vibrios. Appl Microbiol 19: 463-469.
7. Li M, Shimada T, Morris JG, Jr., Sulakvelidze A, Sozhamannan S (2002) Evidence for the emergence of non-O1 and non-O139 *Vibrio cholerae* strains with pathogenic potential by exchange of O-antigen biosynthesis regions. Infect Immun 70: 2441--2453.
8. Meibom KL, Blokesch M, Dolganov NA, Wu C-Y, Schoolnik GK (2005) Chitin induces natural competence in *Vibrio cholerae*. Science 310: 1824-1827.
9. Zhu J, Miller MB, Vance RE, Dziejman M, Bassler BL, et al. (2002) Quorum-sensing regulators control virulence gene expression in *Vibrio cholerae*. Proc Natl Acad Sci USA 99: 3129-3134.
10. Joelsson A, Liu Z, Zhu J (2006) Genetic and phenotypic diversity of quorum-sensing systems in clinical and environmental isolates of *Vibrio cholerae*. Infect Immun 74: 1141-1147.
