# Supplementary material for: Serogroup Conversion of Vibrio cholerae in Aquatic Reservoirs
Source: PLoS Pathog. 2007 Jun 8;3(6):e81. doi: 10.1371/journal.ppat.0030081 (PMC1891326; doi:10.1371/journal.ppat.0030081)
Supplement: Supplementary file 3 [file ppat.0030081.sg002.pdf]

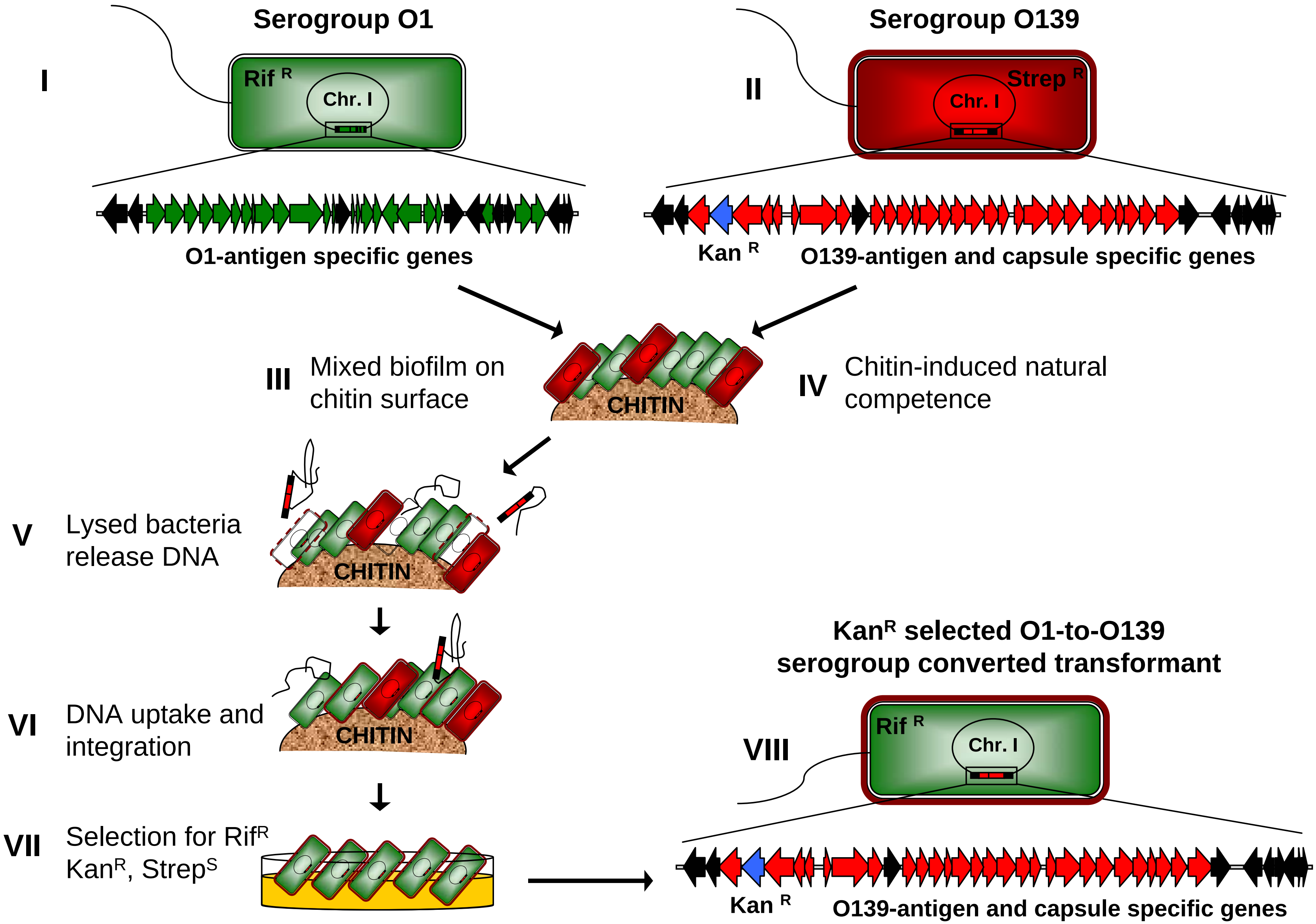

Serogroup O1
I
Rif R
Chr. I
O1-antigen specific genes
Serogroup O139
II
Strep R
Chr. I
Kan R
O139-antigen and capsule specific genes
III
Mixed biofilm on chitin surface
CHITIN
IV
Chitin-induced natural competence
V
Lysed bacteria release DNA
CHITIN
VI
DNA uptake and integration
CHITIN
VII
Selection for RifR KanR, StrepS
KanR selected O1-to-O139 serogroup converted transformant
VIII
Rif R
Chr. I
Kan R
O139-antigen and capsule specific genes

Fig. S2
