# Supplementary material for: Serogroup Conversion of Vibrio cholerae in Aquatic Reservoirs
Source: PLoS Pathog. 2007 Jun 8;3(6):e81. doi: 10.1371/journal.ppat.0030081 (PMC1891326; doi:10.1371/journal.ppat.0030081)
Supplement: Supplementary file 4 [file ppat.0030081.sg003.pdf]

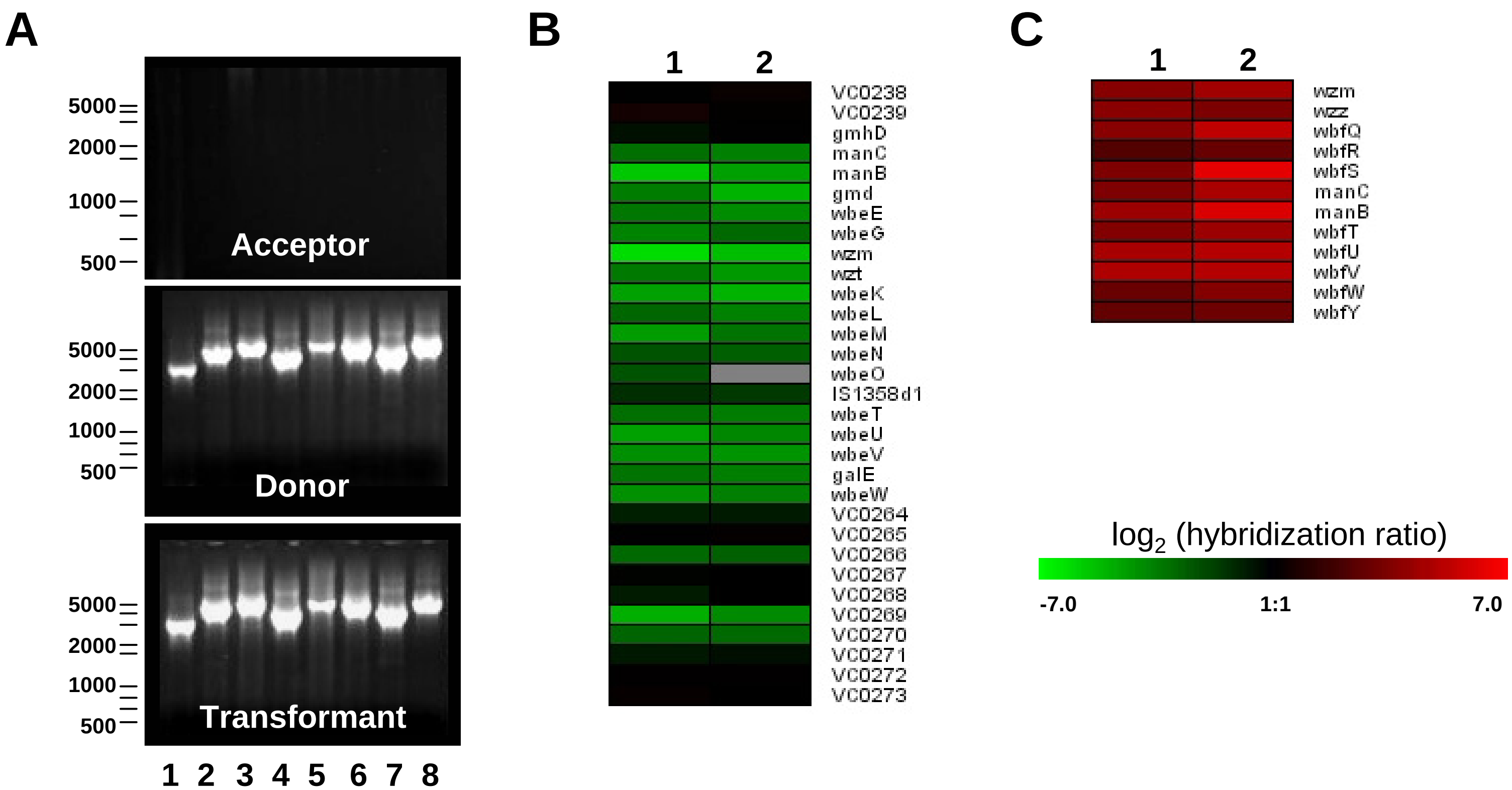
A
5000
2000
1000
500
Acceptor
Donor
Transformant
1 2 3 4 5 6 7 8
B
1 2
VC0238
VC0239
gmhD
manC
manB
gmd
wbeE
wbeG
wzm
wzt
wbeK
wbeL
wbeM
wbeN
wbeO
IS1358d1
wbeT
wbeU
wbeV
galE
wbeW
VC0264
VC0265
VC0266
VC0267
VC0268
VC0269
VC0270
VC0271
VC0272
VC0273
C
1 2
wzm
wzz
wbfQ
wbfR
wbfS
manC
manB
wbfT
wbfU
wbfV
wbfW
wbfY
log2 (hybridization ratio)
-7.0
1:1
7.0

Fig. S3
