# Supplementary material for: Serogroup Conversion of Vibrio cholerae in Aquatic Reservoirs
Source: PLoS Pathog. 2007 Jun 8;3(6):e81. doi: 10.1371/journal.ppat.0030081 (PMC1891326; doi:10.1371/journal.ppat.0030081)
Supplement: Supplementary file 5 [file ppat.0030081.sg004.pdf]

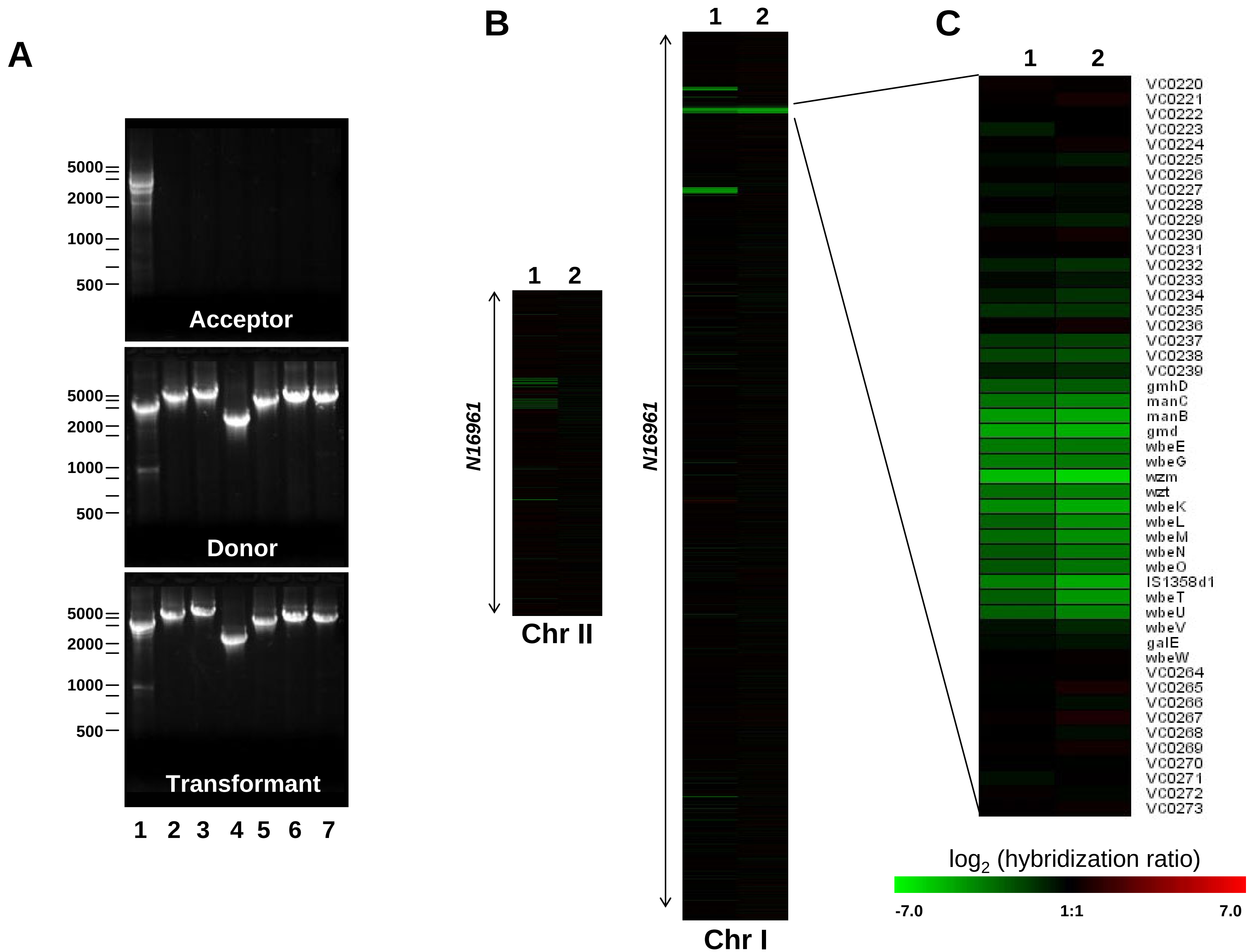
A
5000
2000
1000
500
Acceptor
5000
2000
1000
500
Donor
5000
2000
1000
500
Transformant
1 2 3 4 5 6 7
B
1 2
N16961
Chr II
1 2
N16961
Chr I
C
1 2
VC0220
VC0221
VC0222
VC0223
VC0224
VC0225
VC0226
VC0227
VC0228
VC0229
VC0230
VC0231
VC0232
VC0233
VC0234
VC0235
VC0236
VC0237
VC0238
VC0239
gmhD
manC
manB
gmd
wbeE
wbeG
wzm
wzt
wbeK
wbeL
wbeM
wbeN
wbeO
IS1358d1
wbeT
wbeU
wbeV
galE
wbeW
VC0264
VC0265
VC0266
VC0267
VC0268
VC0269
VC0270
VC0271
VC0272
VC0273
log2 (hybridization ratio)
-7.0
1:1
7.0

Fig. S4
