# Supplementary material for: Serogroup Conversion of Vibrio cholerae in Aquatic Reservoirs
Source: PLoS Pathog. 2007 Jun 8;3(6):e81. doi: 10.1371/journal.ppat.0030081 (PMC1891326; doi:10.1371/journal.ppat.0030081)
Supplement: Supplementary file 6 [file ppat.0030081.sg005.pdf]

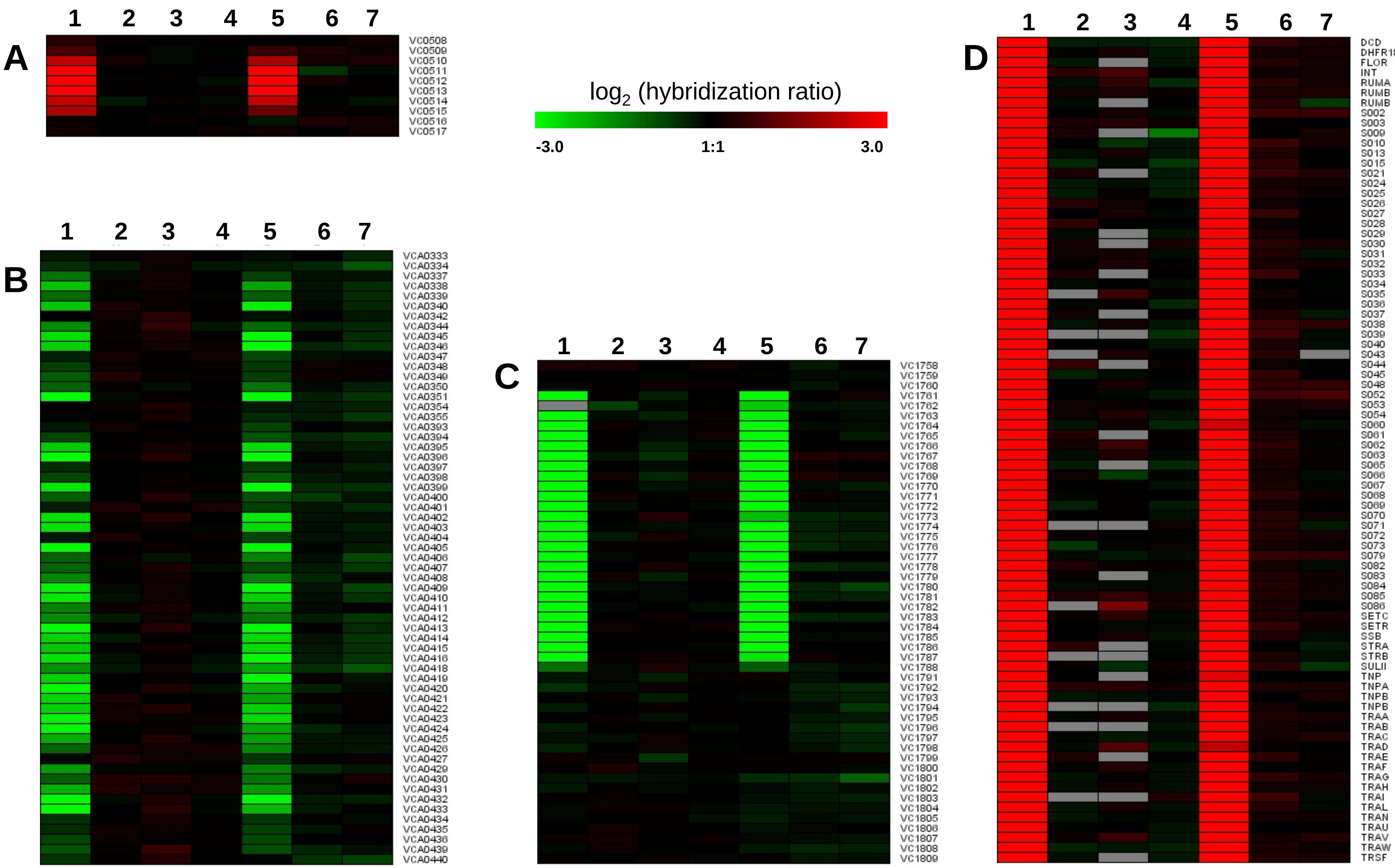
A
B
C
D
1 2 3 4 5 6 7
log2 (hybridization ratio)
-3.0
1:1
3.0
VC0508
VC0509
VC0510
VC0511
VC0512
VC0513
VC0514
VC0515
VC0516
VC0517
VCA0333
VCA0334
VCA0337
VCA0338
VCA0339
VCA0340
VCA0342
VCA0344
VCA0345
VCA0346
VCA0347
VCA0348
VCA0349
VCA0350
VCA0351
VCA0354
VCA0355
VCA0393
VCA0394
VCA0395
VCA0396
VCA0397
VCA0398
VCA0399
VCA0400
VCA0401
VCA0402
VCA0403
VCA0404
VCA0405
VCA0406
VCA0407
VCA0408
VCA0409
VCA0410
VCA0411
VCA0412
VCA0413
VCA0414
VCA0415
VCA0416
VCA0418
VCA0419
VCA0420
VCA0421
VCA0422
VCA0423
VCA0424
VCA0425
VCA0426
VCA0427
VCA0429
VCA0430
VCA0431
VCA0432
VCA0433
VCA0434
VCA0435
VCA0436
VCA0439
VCA0440
VC1758
VC1759
VC1760
VC1761
VC1762
VC1763
VC1764
VC1765
VC1766
VC1767
VC1768
VC1769
VC1770
VC1771
VC1772
VC1773
VC1774
VC1775
VC1776
VC1777
VC1778
VC1779
VC1780
VC1781
VC1782
VC1783
VC1784
VC1785
VC1786
VC1787
VC1788
VC1791
VC1792
VC1793
VC1794
VC1795
VC1796
VC1797
VC1798
VC1799
VC1800
VC1801
VC1802
VC1803
VC1804
VC1805
VC1806
VC1807
VC1808
VC1809
DCD
DHFR18
FLOR
INT
RUMA
RUMB
RUMB
S002
S003
S009
S010
S013
S015
S021
S024
S025
S026
S027
S028
S029
S030
S031
S032
S033
S034
S035
S036
S037
S038
S039
S040
S043
S044
S045
S048
S052
S053
S054
S060
S061
S062
S063
S065
S066
S067
S068
S069
S070
S071
S072
S073
S079
S082
S083
S084
S085
S086
SETC
SETR
SSB
STRA
STRB
SULII
TNP
TNPA
TNPB
TNPB
TRAA
TRAB
TRAC
TRAD
TRAE
TRAF
TRAG
TRAH
TRAI
TRAL
TRAN
TRAU
TRAV
TRAW
TRSF

**Fig. S5**
