# Supplementary material for: Serogroup Conversion of Vibrio cholerae in Aquatic Reservoirs
Source: PLoS Pathog. 2007 Jun 8;3(6):e81. doi: 10.1371/journal.ppat.0030081 (PMC1891326; doi:10.1371/journal.ppat.0030081)
Supplement: Supplementary file 7 [file ppat.0030081.st001.pdf]

Table S1: Oligonucleotides used in this study

Primers	Sequence
Primers used for checking the O139 specific region*	
PCR1A	5'-TTGCTGCCAATCATGCCAGCGCCGCC-3'
PCR1B	5'-CCATGTTTGCCGGTGTGGGCAGTGAGC-3'
PCR2A	5'-GCTCACTGCCCACACCGGCAAACATGG-3'
PCR2B	5'-GGCTAAGTGTCCGTAAGATATATCCTTGGG-3'
PCR3A	5'-CCCAAGGATATATCTTACGGACACTTAGCC-3'
PCR3B	5'-CGTCTTGACCAGTTACACCAGTGATAAGGG-3'
PCR4A	5'-CCCTTATCACTGGTGTAACTGGTCAAGACG-3'
PCR4B	5'-CCTGCTCCTCGCTCAACATTTCGGTAATTAATCG-3'
PCR5A	5'-CGATTAATTACCGAAATGTTGAGCGAGGAGCAGG-3'
PCR5B	5'-TATAGAGGAATAACAACTGAGATATCAAGCG-3'
PCR6A	5'-AATGACGCTTGATATCTCAGTTGTTATTCC-3'
PCR6B	5'-CGGCTACCAACACCACCAGACAACACAACAGGGC-3'
PCR7A	5'-GCCCTGTTGTGTTGTCTGGTGGTGTTGGTAGCCG-3'
PCR7B	5'-GCAAGCCCAAAAAGGCCGCGAGAAAATCAATTAAGCG-3'
PCR8A	5'-GCTTAATTGATTTTCTCGCGGCCTTTTTGGGCTTGC-3'
PCR8B	5'-CCTCGGTAAGTACACGCTCACTCTGCTTGGG-3'
Primers used for checking the O37 specific region#	
O37-1A	5'-TTGCTGCCAATCATGCCAGCGCCGCC-3'
O37-1B	5'-CTTCGCCAACTCAATTGGGTGTGATGC-3'
O37-2A	5'-CACCCAATTGAGTTGGCGAAGTCACC-3'
O37-2B	5'-TGAAAAGCCCACGGTTCTAAGGCTCC-3'
O37-3A	5'-CCTTAGAACCGTGGGCTTTTCAGTCG-3'
O37-3B	5'-ATTAATGAGTTCAATTAAGTCATTCCC-3'
O37-4A	5'-GACTTAATTGAACTCATTAATAACTCG-3'
O37-4B	5'-CTATGTTCTACAGCCCACTTGTTAGC-3'
O37-5A	5'-ACTTTAACTCTTGCTAACAAGTGGGC-3'
O37-5B	5'- ACTCCCGCGATAGCTGTTACTGCAGG-3'
O37-6A	5'-ACTCCTGCAGTAACAGCTATCGCGGG-3'

O37-6B	5'-ATGACTTCTTCTTAGTTTTGAATCC-3'
O37-7A	5'-TCAAAACTAAGAAGAAGTCATTTGC-3'
O37-7B	5'-TTCCCTCCGTGATGAACCACTTCC-3'
Primers for the construction of strain VCO139-Kan	
1-wbfB-up-NcoI	5'-GCGCCATGGTGACCCAAGCTCAGCCTTTGGC-3'
2-wbfB-end-OL-Kan	5'-GTAAGCAGACAGTATATTGCTATGCA AAAAATGATCCTAAAGTG-3'
3-kanR-up-OL-wbfB	5'-CATAGCAATATACTGTCTGCTTA CATAAACAGTAATACAAGG-3'
4-kanR-down-OL-wbfA	5'-GCCAAGTTGAGTCCGCTCATGAATT AATTCTTAGAAAAACTCATCG-3'
5-wbfA-start-OL-Kan	5'-ATTCATGAGCGGACTCAACTTG GCAAACTCAGGGACTTTACATCATGC-3'
6-wbfA- down-SacI	5'-CGCGAGCTCACTTGTTTGCGGGTTGCTATTATTGCCG-3'
Primers for the construction of strain VCO139-KanΔIS1358	
1-IS1358-KO-SacI	5'-CGCGAGCTCGATGCAAGAAGCGTATAAAGTGTTTAGC-3'
2-IS1358-KO	5'-TGAACATTACCGTCATGC GGTTTCCCTGACATCGGCATGTTCATG-3'
3-IS1358-KO	5'-GGAAACCGCATGACGGTAAT GTTCATGCGGTTTCCGTGACGTGACGG-3'
4- IS1358-KO-NcoI	5'-GCGCCATGGCATCCACATCCGCTGTGTATTCAGGAG-3'
Primers for the construction of strain O37-Kan	
1-ORF6 -up-*XbaI*	5'-GCGTCTAGATAAGCGAGGGTATAAACTTCATATCC-3'
2-ORF6-end-OL-Kan	5'-GTAAGCAGACAGTTCATGCAG CAGAGCATTATAAATTAACTATAAC-3'
3-*kanR*-up-OL-ORF6	5'-CTGCTGCATGAACTGTCTGCTTACAT AAACAGTAATACAAGG-3'
4-*kanR*-down-OL-ORF7	5'-TACTGCTTACATTCCGCTCATGAATTAAT TCTTAGAAAAACTCATCG-3'
5-ORF7-start-OL-	5'-ATTCATGAGCGGAATGTAAGCAGT

Kan	ATTAGATACAAAGAGTTTGG-3’
6-ORF7-*down-Sac*I	5’-CGCGAGCTCGTTTTAGTGTGCTCAAGATTTCGG-3’

* Primer PCR1A – 8B correspond to Fig. 1; from left to right

Primer O37-1A – 7B were designed based on the O37 sequence of Li *et al*. [7]

Supporting references:

1. Waldor MK, Mekalanos JJ (1994) *Vibrio cholerae* O139 specific gene sequences. Lancet 343: 1366.
2. Waldor MK, Mekalanos JJ (1994) ToxR regulates virulence gene expression in non-O1 strains of *Vibrio cholerae* that cause epidemic cholera. Infect Immun 62: 72-78.
3. Meibom KL, Li XB, Nielsen AT, Wu CY, Roseman S, et al. (2004) The *Vibrio cholerae* chitin utilization program. Proc Natl Acad Sci USA 101: 2524-2529.
4. Fullner KJ, Mekalanos JJ (1999) Genetic characterization of a new type IV-A pilus gene cluster found in both classical and El Tor biotypes of *Vibrio cholerae.* Infect Immun 67: 1393-1404.
5. Aldova E, Laznickova K, Stepankova E, Lietava J (1968) Isolation of nonagglutinable vibrios from an enteritis outbreak in Czechoslovakia. J Infect Dis 118: 25-31.
6. Felsenfeld O, Stegherr-Barrios A, Aldova E, Holmes J, Parrott MW (1970) In vitro and in vivo studies of streptomycin-dependent cholera vibrios. Appl Microbiol 19: 463-469.
7. Li M, Shimada T, Morris JG, Jr., Sulakvelidze A, Sozhamannan S (2002) Evidence for the emergence of non-O1 and non-O139 *Vibrio cholerae* strains with pathogenic potential by exchange of O-antigen biosynthesis regions. Infect Immun 70: 2441-2453.
8. Meibom KL, Blokesch M, Dolganov NA, Wu C-Y, Schoolnik GK (2005) Chitin induces natural competence in *Vibrio cholerae*. Science 310: 1824-1827.
9. Zhu J, Miller MB, Vance RE, Dziejman M, Bassler BL, et al. (2002) Quorum-sensing regulators control virulence gene expression in *Vibrio cholerae*. Proc Natl Acad Sci USA 99: 3129-3134.
10. Joelsson A, Liu Z, Zhu J (2006) Genetic and phenotypic diversity of quorum-sensing systems in clinical and environmental isolates of *Vibrio cholerae*. Infect Immun 74: 1141-1147.
11. Heidelberg JF, Eisen JA, Nelson WC, Clayton RA, Gwinn ML, et al. (2000) DNA sequence of both chromosomes of the cholera pathogen *Vibrio cholerae*. Nature 406: 477-483.
12. Mazel D, Dychinco B, Webb VA, Davies J (1998) A distinctive class of integron in the *Vibrio cholerae* genome. Science 280: 605-608.
13. Jermyn WS, Boyd EF (2002) Characterization of a novel Vibrio pathogenicity island (VPI-2) encoding neuraminidase (nanH) among toxigenic Vibrio cholerae isolates. Microbiology 148: 3681-3693.
14. Dziejman M, Balon E, Boyd D, Fraser CM, Heidelberg JF, et al. (2002) Comparative genomic analysis of *Vibrio cholerae*: genes that correlate with cholera endemic and pandemic disease. Proc Natl Acad Sci USA 99: 1556-1561.
15. Beaber JW, Hochhut B, Waldor MK (2002) Genomic and functional analyses of SXT, an integrating antibiotic resistance gene transfer element derived from *Vibrio cholerae*. J Bacteriol 184: 4259-4269.
