# Supplementary material for: Serogroup Conversion of Vibrio cholerae in Aquatic Reservoirs
Source: PLoS Pathog. 2007 Jun 8;3(6):e81. doi: 10.1371/journal.ppat.0030081 (PMC1891326; doi:10.1371/journal.ppat.0030081)
Supplement: Supplementary file 8 [file ppat.0030081.st002.pdf]

Table S2: List of microarrays and corresponding GEO accession numbers.

GEO Samples accession number	Cy3 labelled	Cy5 labelled	Description	GEO Series accession number*
O1 → O139 Serogroup conversion / Kan^R selected				
GSM115549	A1552	VCO139	CA1108 - DNA; A1552 vs. VCO139-Kan	GSE5120
GSM115571	A1552	VCO139	CA1109 - DNA; A1552 vs. VCO139-Kan	GSE5120
GSM115560	A1552	VCO139	CA1122 - DNA; A1552 vs. VCO139-Kan	GSE5120
GSM115562	A1552	VCO139	CA1123 - DNA; A1552 vs. VCO139-Kan	GSE5120
GSM115587	A1552	VCO139	CA1067 - DNA; A1552 vs. VCO139-Kan	GSE5120
GSM115589	A1552	VCO139	CA1068 - DNA; A1552 vs. VCO139-Kan	GSE5120
GSM115573	A1552	ASGC#1	CA1110 - DNA; A1552 vs. ASGC#1	GSE5120
GSM115575	A1552	ASGC#1	CA1111 - DNA; A1552 vs. ASGC#1	GSE5120
GSM115577	A1552	ASGC#2	CA1112 - DNA; A1552 vs. ASGC#2	GSE5120
GSM115579	A1552	ASGC#2	CA1113 - DNA; A1552 vs. ASGC#2	GSE5120
GSM115582	A1552	ASGC#3	CA1114 - DNA; A1552 vs. ASGC#3	GSE5120
GSM115585	A1552	ASGC#3	CA1115 - DNA; A1552 vs. ASGC#3	GSE5120
GSM115564	A1552	AIISGC#1	CA1124 - DNA; A1552 vs. AIISGC#1	GSE5120
GSM115566	A1552	AIISGC#1	CA1125 - DNA; A1552 vs. AIISGC#1	GSE5120
GSM115572	A1552	AIISGC#2	CA1126 - DNA; A1552 vs. AIISGC#2	GSE5120
GSM115574	A1552	AIISGC#2	CA1127 - DNA; A1552 vs. AIISGC#2	GSE5120
GSM115576	A1552	AIISGC#3	CA1128 - DNA; A1552 vs. AIISGC#3	GSE5120
GSM115578	A1552	AIISGC#3	CA1129 - DNA; A1552 vs. AIISGC#3	GSE5120
GSM115555	A1552	AIIISGC#1	CA1136 - DNA; A1552 vs. AIIISGC#1	GSE5120

GSM115557	A1552	AIIISGC#1	CA1137 - DNA; A1552 vs. AIIISGC#1	GSE5120
GSM115559	A1552	AIIISGC#2	CA1057- DNA; A1552 vs. AIIISGC#2	GSE5120
GSM115561	A1552	AIIISGC#2	CA1058 - DNA; A1552 vs. AIIISGC#2	GSE5120
GSM115563	A1552	AIIISGC#3	CA1059 - DNA; A1552 vs. AIIISGC#3	GSE5120
GSM115565	A1552	AIIISGC#3	CA1060 - DNA; A1552 vs. AIIISGC#3	GSE5120
O1 → O139 serogroup conversion / mixed biofilms				
GSM138055	A1552	A1552SGC-A	CB093-A1552 vs. A1552SGC-A	GSE5932
GSM138056	A1552	A1552SGC-A	CB094-A1552 vs. A1552SGC-A	GSE5932
GSM138057	A1552	A1552SGC-B	CB095-A1552 vs. A1552SGC-B	GSE5932
GSM138058	A1552	A1552SGC-B	CB096-A1552 vs. A1552SGC-B	GSE5932
GSM138059	A1552	A1552SGC-C	CB097-A1552 vs. A1552SGC-C	GSE5932
GSM138060	A1552	A1552SGC-C	CB098-A1552 vs. A1552SGC-C	GSE5932
GSM138068	A1552	MCISGC#1	CA1093-A1552 vs. MCISGC#1	GSE5932
GSM138069	A1552	MCISGC#1	CA1095-A1552 vs. MCISGC#1	GSE5932
GSM138070	A1552	MCIISGC#1	CB057-A1552 vs. MCIISGC#1	GSE5932
GSM138063	A1552	MCIISGC#1	CB058-A1552 vs. MCIISGC#1	GSE5932
GSM138066	A1552	MCIISGC#2	CA1075-A1552 vs. MCIISGC#2	GSE5932
GSM138067	A1552	MCIISGC#2	CA1076-A1552 vs. MCIISGC#2	GSE5932
GSM138064	A1552	MCIIISGC#1	CB059-A1552 vs. MCIIISGC#1	GSE5932
GSM138065	A1552	MCIIISGC#1	CB060-A1552 vs. MCIIISGC#1	GSE5932
GSM138061	A1552	MCIIISGC#2	CB501-A1552 vs. MCIIISGC#2	GSE5932
GSM138062	A1552	MCIIISGC#2	CB502-A1552 vs. MCIIISGC#2	GSE5932
O1 → O37 serogroup conversion / phage selected				
GSM138090	A1552	ATCC25872	CB503-DNA-A1552 vs. ATCC25872	GSE5934
GSM138092	A1552	ATCC25872	CB504-DNA-A1552 vs. ATCC25872	GSE5934
GSM138096	A1552	ATCC25872	CB509-DNA-A1552 vs. ATCC25872	GSE5934
GSM138094	A1552	AIpO37#1	CB515- DNA; A1552 vs. AIpO37#1	GSE5934
GSM138095	A1552	AIpO37#1	CB516- DNA; A1552 vs. AIpO37#1	GSE5934
GSM138097	A1552	AIpO37#4	CB517- DNA; A1552 vs. AIpO37#4	GSE5934
GSM138099	A1552	AIpO37#4	CB518- DNA; A1552 vs. AIpO37#4	GSE5934
GSM138101	A1552	AIpO37#6	CB519- DNA; A1552 vs. AIpO37#6	GSE5934
GSM138103	A1552	AIpO37#6	CB520- DNA; A1552 vs.	GSE5934

			AIpO37#6	
GSM138105	A1552	AIpO37#8	CB521- DNA; A1552 vs. AIpO37#8	GSE5934
GSM138093	A1552	AIpO37#8	CB522- DNA; A1552 vs. AIpO37#8	GSE5934
GSM138107	A1552	AIIpO37#9	CB499- DNA; A1552 vs. AIIpO37#9	GSE5934
GSM138109	A1552	AIIpO37#9	CB500- DNA; A1552 vs. AIIpO37#9	GSE5934
GSM138112	A1552	AIIpO37#13	CB531- DNA; A1552 vs. AIIpO37#13	GSE5934
GSM138098	A1552	AIIpO37#13	CB532- DNA; A1552 vs. AIIpO37#13	GSE5934
GSM138102	A1552	AIIpO37#16	CB534- DNA; A1552 vs. AIIpO37#16	GSE5934
GSM138100	A1552	AIIpO37#16	CB533- DNA; A1552 vs. AIIpO37#16	GSE5934
GSM138104	A1552	AIIIpO37#9A	CB535- DNA; A1552 vs. AIIIpO37#9A	GSE5934
GSM138106	A1552	AIIIpO37#9A	CB536- DNA; A1552 vs. AIIIpO37#9A	GSE5934
GSM138111	A1552	AIVpO37#1A	CB524- DNA; A1552 vs. AIVpO37#1A	GSE5934
GSM138113	A1552	AIVpO37#1A	CB525- DNA; A1552 vs. AIVpO37#1A	GSE5934
GSM138114	A1552	AIVpO37#3A	CB526- DNA; A1552 vs. AIVpO37#3A	GSE5934
GSM138115	A1552	AIVpO37#3A	CB527- DNA; A1552 vs. AIVpO37#3A	GSE5934
GSM138087	A1552	AIVpO37#4A	CB528- DNA; A1552 vs. AIVpO37#4A	GSE5934
GSM138088	A1552	AIVpO37#4A	CB529- DNA; A1552 vs. AIVpO37#4A	GSE5934
GSM138089	A1552	AIVpO37#8A	CB530- DNA; A1552 vs. AIVpO37#8A	GSE5934
GSM138091	A1552	AIVpO37#8A	CB270- DNA; A1552 vs. AIVpO37#8A	GSE5934
O1 → O139 serogroup conversion / phage selected				
GSM120146	A1552	MO10	CB479- DNA; A1552 vs. MO10	GSE5302
GSM120148	A1552	MO10	CB480- DNA; A1552 vs. MO10	GSE5302
GSM120157	A1552	ApO139#2	CB451- DNA; A1552 vs. ApO139#2	GSE5302
GSM120159	A1552	ApO139#2	CB452- DNA; A1552 vs. ApO139#2	GSE5302
GSM120163	A1552	ApO139#4	CB456- DNA; A1552 vs.	GSE5302

			ApO139#4	
GSM120164	A1552	ApO139#4	CB457- DNA; A1552 vs. ApO139#4	GSE5302
GSM120156	A1552	ApO139#6	CB459- DNA; A1552 vs. ApO139#6	GSE5302
GSM120165	A1552	ApO139#6	CB458- DNA; A1552 vs. ApO139#6	GSE5302
GSM120158	A1552	ApO139#8	CB461- DNA; A1552 vs. ApO139#8	GSE5302
GSM120160	A1552	ApO139#8	CB462- DNA; A1552 vs. ApO139#8	GSE5302
GSM120161	A1552	MO10	CB454- DNA; A1552 vs. MO10	GSE5302
GSM120162	A1552	MO10	CB455- DNA; A1552 vs. MO10	GSE5302
GSM120147	A1552	AIIpO139#3	CB471- DNA; A1552 vs. AIIpO139#3	GSE5302
GSM120149	A1552	AIIpO139#3	CB472- DNA; A1552 vs. AIIpO139#3	GSE5302
GSM120150	A1552	AIIpO139#4	CB473- DNA; A1552 vs. AIIpO139#4	GSE5302
GSM120151	A1552	AIIpO139#4	CB474- DNA; A1552 vs. AIIpO139#4	GSE5302
GSM120152	A1552	AIIpO139#5	CB475- DNA; A1552 vs. AIIpO139#5	GSE5302
GSM120153	A1552	AIIpO139#5	CB476- DNA; A1552 vs. AIIpO139#5	GSE5302
GSM120154	A1552	AIIpO139#6	CB477- DNA; A1552 vs. AIIpO139#6	GSE5302
GSM120155	A1552	AIIpO139#6	CB478- DNA; A1552 vs. AIIpO139#6	GSE5302
GSM138086	A1552	AIIIpO139#1	CB481- DNA; A1552 vs. AIIIpO139#1	GSE5302
GSM138076	A1552	AIIIpO139#1	CB482- DNA; A1552 vs. AIIIpO139#1	GSE5302
GSM138078	A1552	AIIIpO139#3	CB483- DNA; A1552 vs. AIIIpO139#3	GSE5302
GSM138080	A1552	AIIIpO139#3	CB484- DNA; A1552 vs. AIIIpO139#3	GSE5302
GSM138082	A1552	AIIIpO139#4	CB485- DNA; A1552 vs. AIIIpO139#4	GSE5302
GSM138083	A1552	AIIIpO139#4	CB486- DNA; A1552 vs. AIIIpO139#4	GSE5302
GSM138084	A1552	AIIIpO139#5	CB487- DNA; A1552 vs. AIIIpO139#5	GSE5302
GSM138085	A1552	AIIIpO139#5	CB488- DNA; A1552 vs. AIIIpO139#5	GSE5302
GSM138073	A1552	AIVpO139#2	CB489- DNA; A1552 vs.	GSE5302

			AIVpO139#2	
GSM138074	A1552	AIVpO139#2	CB490- DNA; A1552 vs. AIVpO139#2	GSE5302
GSM138071	A1552	AIVpO139#4	CB450- DNA; A1552 vs. AIVpO139#4	GSE5302
GSM138072	A1552	AIVpO139#4	CB460- DNA; A1552 vs. AIVpO139#4	GSE5302
GSM138075	A1552	AIVpO139#5	CB492- DNA; A1552 vs. AIVpO139#5	GSE5302
GSM138077	A1552	AIVpO139#5	CB493- DNA; A1552 vs. AIVpO139#5	GSE5302
GSM138079	A1552	AIVpO139#8	CB494- DNA; A1552 vs. AIVpO139#8	GSE5302
GSM138081	A1552	AIVpO139#8	CB495- DNA; A1552 vs. AIVpO139#8	GSE5302

***** The microarray data have been deposited in the NIH National Center for Biotechnology Information’s Gene Expression Omnibus (GEO, www.ncbi.nlm.nih.gov/geo/).
